# The Properties and Microstructure of Na_2_CO_3_ and Al-10Sr Alloy Hybrid Modified LM6 Using Ladle Metallurgy Method

**DOI:** 10.3390/ma16206780

**Published:** 2023-10-20

**Authors:** Mhd Noor Ervina Efzan, Hao Jie Kong

**Affiliations:** Faculty of Engineering and Technology (FET), Multimedia University (MMU), Ayer Keroh 5450, Malaysia; khj_haojie@hotmail.com

**Keywords:** ladle, aluminium, LM6, microstructure, alloy

## Abstract

In this work, Al-10Sr alloy and Na_2_CO_3_ were added to LM6 (reference alloy) as hybrid modifiers through ladle metallurgy. The microstructure enhancement was analyzed using an optical microscope (OM). The results were further confirmed with Scanning Electron Microscope (SEM) and Energy Dispersive X-ray (EDX) spectroscopy. The results showed that Na_2_CO_3_ and Al-10Sr alloy successfully hybrid modified the sharp needle-like eutectic Si into fibrous eutectic Si. Soft primary Al dendrites were also discovered after the hybrid modification. The formation of β-Fe flakes was suppressed, and α-Fe sludge was transformed into Chinese script morphology. A 2.13% density reduction was recorded. A hardness test was also performed to investigate the mechanical improvement of the hybrid-modified LM6. 2.3% of hardness reduction was recorded in the hybrid-modified LM6 through ladle metallurgy. Brittle cracks were not observed, while ductile pile-ups were the main features that appeared on the indentations of hybrid-modified LM6, indicating a brittle to ductile transformation after hybrid modification of LM6 by Na_2_CO_3_ and Al-10Sr alloy through ladle metallurgy.

## 1. Introduction

Aluminium-Silicon (Al-Si) alloy has been widely used as a lightweight material in aerospace and automotive industries [1,2,3,4,5,6]. However, Al-Si alloy is brittle due to the sharp needle-like Si morphology in the microstructure [7,8]. Efforts have been made recently to reduce the brittleness of Al-Si alloy through different techniques such as the alloying method, controlling the overheat temperature and cooling rate, performing iso-thermal mechanical stirring during the semi-solidified state, and the incorporation of ultrasonic vibration during the solidification process [9,10,11]. Researchers found out that the addition of modifiers such as Sodium (Na) and Strontium (Sr) can transform the needle-like Silicon (Si) into fibrous-like Si morphology [12,13]. Owing to the demanding requirements of the industries, Lu et al. [12] proposed that the modification solely based on a single type of modifier is unsatisfactory. For instance, Na is a fast-acting modifier, able to produce an immediate modification on the Si morphology.

Nevertheless, due to its high reactivity, its potency as an effective modifier diminishes over a long melting period. On the other hand, Sr can remedy its deficiency by serving as a slow-acting modifier with more pronounced modification power over a longer melt-holding period. Therefore, the combined addition of modifiers (hybrid modification) is considered important.

Na and Sr are very reactive. They oxidize rapidly when exposed to the atmosphere, especially at an elevated temperature. Thus, Na and Sr are seldom added as pure elements. Sr is normally added as an Al-Sr system alloy [12,13]. Meanwhile, Na is usually added as an Al-Si-Na system alloy [13] or Sodium Floride (NaF) [12]. Sodium Carbonate (Na_2_CO_3_) has been widely used as a flux in treating insoluble silicates. However, its potential to serve as a modifying flux in Al-Si alloy is not reported. Conventionally, modifiers are added inside the melting furnace [14]. Therefore, a long melt-holding period is required for hybrid modifiers’ complete dissolution and homogenization. The prolonged melt holding period and high melting temperature in the melting furnace significantly reduce the availability of potent modifiers due to rapid oxidization. Thus, hybrid modifiers will be added in a ladle (ladle metallurgy) in this study. Dube et al. [15] developed a methodology to produce aluminium alloy via a ladle metallurgical process. According to the authors, ladle metallurgy can reduce the risk of contamination, especially if two or more alloys are produced. Besides, a well-insulated ladle can allow alloying operation to be carried out without any external heat input, which is particularly important during the alloying of Magnesium (Mg), Copper (Cu), and Silicon (Si) that involves endothermic dissolution.

Despite the early introduction of ladle metallurgy in the smelting of aluminium alloys, scarce study has been reported on the addition of modifiers and grain refiners through ladle metallurgy. The most recent study of modification through ladle metallurgy in aluminium alloys can be dated back to 2006, reported by Pena and Lozano [7]. In that study, additional modifiers Ti and Sr improve mechanical performance after the modification. Scarce literature on ladle metallurgy might be due to the preference for stream inoculation in the aluminium smelting industry. In-stream inoculation, modifiers and grain refiners are added before molten metal enters the casting moulds [16,17]. The addition is made in the launders between treatment vessels and casting stations. Through this method, the fading of modifiers and grain refiners can be reduced. Even though stream inoculation improves the modification and grain refinement of aluminium alloys, the modifiers and grain refiners should possess a high dissolution rate so that complete dissolution can be achieved. Besides, the pouring temperature of the molten metal should be high enough for the melt to flow smoothly through the launder. Higher pouring temperature will lead to the oxidation of modifiers and grain refiners. To solve these issues, the addition of hybrid modifiers through ladle metallurgy will be studied in this work.

This work aims to determine the influences of Al-Sr alloy and Na_2_CO_3_ as hybrid modifiers in density, microstructure, and hardness of LM6 alloy via ladle metallurgy.

## 2. Experimental Works

LM6 alloy, Al-10Sr alloy, and anhydrous Na_2_CO_3_ (99.5% pure) powder were used to prepare the hybrid modified Al-12Si-0.02Sr-0.02Na system alloy. The LM6 alloy was in the form of an ingot, conforming to the British Standards 1490 [18]. The LM6 alloy and Al-10Sr alloy were purchased from FES Foundry Equipment Supply (M) Sdn. Bhd. while the Na_2_CO_3_ powder was acquired from Comak Chemical Limited.

**Figure 1:**
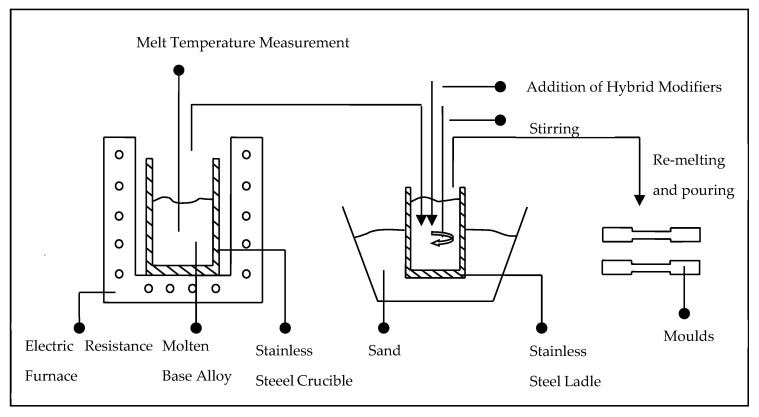
A schematic showing the ladle metallurgy melting sequence.

**Figure 2:**
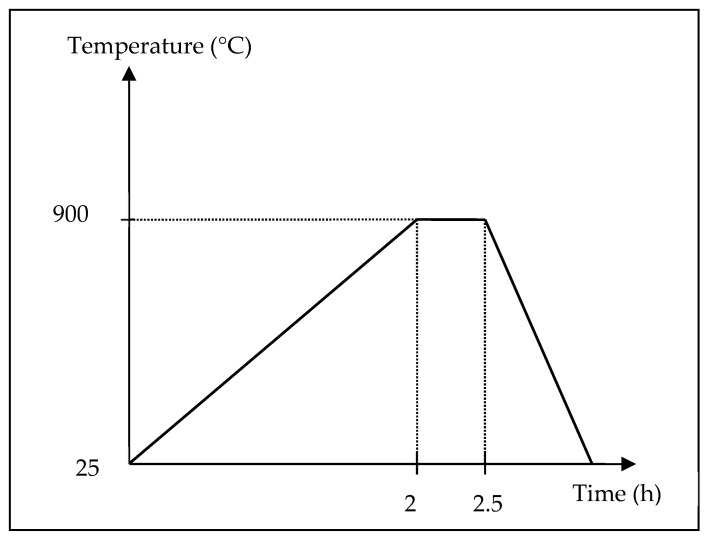
Temperature profile of the melting process.

Before melting, small pieces of LM6 were cut with a dimension of 2.5 cm^3^. Acetone was used to remove the dirt and impurities thoroughly. A digital weighing balance, Dragon 303, Mettler Toledo, was used to measure the mass of the cut LM6 and hybrid modifiers. Ladle metallurgy, or mixing alloying elements in a ladle [7], was incorporated into the melting process, as shown in Figure 1. LM6 was melted in an electrical resistance furnace, K 4/13, Nabertherm, according to the temperature profile in Figure 2. The melt was brought to a melting temperature of 900 °C in 2 h and soaked for another 30 min for homogenization. Ladle metallurgy was performed in the following sequence: charging hybrid modifiers in the ladle, followed by pouring molten LM 6 from the furnace into the ladle after slag skimming and stirring for 10 s. The melt containing molten LM 6 and hybrid modifiers in the ladle was immediately stirred for 5 s. Afterwards, re-melt was performed for 15 min at 900 °C to enhance the compositional homogenization of the alloy. The melting ended with the casting process, where the re-melted alloy was poured into a preheated (100 °C) metallic (ASSAB 8407) mold and left for solidification.

Samples were ground using silicon carbide papers and polished to a mirror-like surface to remove scratches and artefacts. The density of the samples was measured using an electronic densimeter, MD-300S, Alta Mirage. For metallurgical analysis, etching was performed using Keller Reagent for 5 seconds to reveal the microstructure. Microstructure observation was performed under a reflected high-power optical microscope, Meiji Techno Japan, equipped with bright field imaging. The results were further analyzed under a scanning electron microscope (SEM), EVO 50, Carl Zeiss AG, in secondary electron (SE) mode. Energy Dispersive X-ray (EDX) was also performed at 1 k magnification to confirm the elements in the microstructure.

Vickers hardness was performed by applying a load of 2 kg with 15 s of dwell time to evaluate the hardness of the samples. An average hardness was obtained by averaging the hardness gathered from five indentations.

## 3. Results and Discussions

**Density.** Density is one of the important parameters during the selection of materials. Light (low-density) yet high-strength materials are often desirable in the aerospace and automotive industries. The unmodified LM6 recorded a density of 2.717 g/cm^3^. The hybrid-modified LM6 achieved a density of 2.659 g/cm^3^, equivalent to 2.13% reduction. The reduced density of LM6 after the combined addition of Na_2_CO_3_ and Al-Sr alloy can be owing to the formation of porosity. The addition of Sr has often been associated with the formation of porosity in Al-Si alloys [19]. The pores expand due to the lowered pressure and result in a much more porous sample than under atmospheric conditions of solidification.

**Figure 3:**
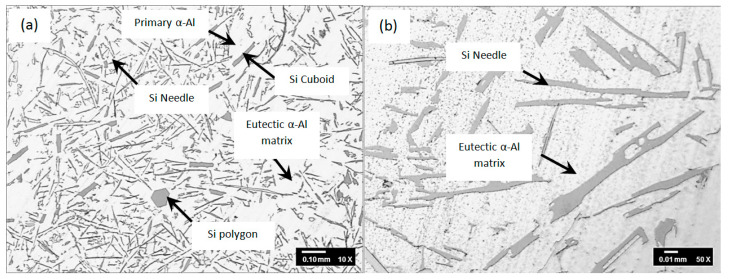
Microstructures of unmodified LM6 through ladle metallurgy at (**a**) 10× and (**b**) 50×.

**Figure 4:**
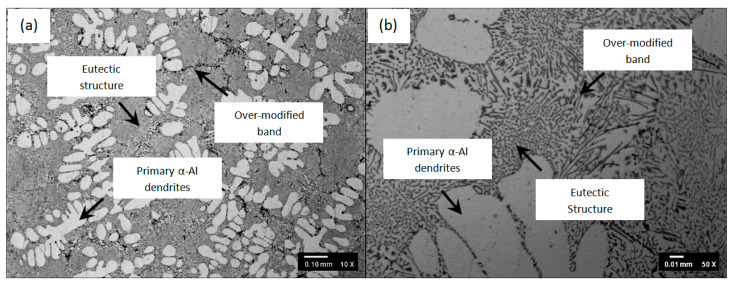
Microstructures of hybrid modified LM6 with Al-Sr alloy and Na_2_CO_3_ and through ladle metallurgy at (**a**) 10× and (**b**) 50×.

**Microstructure. **Figure 3 and Figure 4 show the microstructure of unmodified LM6 and the Al-12Si-0.02Sr-0.02Na system alloy (hybrid modified LM6) at 10 and 50 magnifications.

In the absence of a hybrid modifier, the unmodified LM6 consists of Al-rich (bright zone) and Si-rich (dark zone) phases, as demonstrated in Figure 3. The dark Si-rich phase resembles itself in needle, cuboid, and irregular polygon morphologies. The Si-rich needles combine with the Al-rich phase, forming a eutectic structure consisting of alternating layers of Si-rich needle-like lamellar with an Al-rich matrix. The Si needles have significantly been blamed as stress concentrators that result in the brittleness of the Al-Si alloys [8,20]. On the other hand, the Si-rich cuboids are suggested to be the primary Si phase. This is supported by Pena and Lozano [7]. In their work, they reported that these primary Si-rich cuboids nucleate the primary α-Al phase on some occasions.

After the addition of Na_2_CO_3_ and Al-Sr alloy to the LM6, bright dendrites are observed in Figure 4. However, these dendrites do not appear in the unmodified LM6. They have a similar morphology as the primary Al phase reported in a study on the modification of Al-10Si alloy with 0.0025% Sr carried out by Sun et al. [21]. It is, therefore, a posteriori to deduce that the bright dendrites are indeed the primary Al phase. Another discernible feature that results from the hybrid modification will be the fine black dot dots embedded in the bright Al-rich phase, as shown in Figure 3b. These fine dot-dots were also discovered by Lu et al. [12] in a Na-Sr modified Al-10Si alloy. The authors regarded these dot-dots as modified eutectic Si phase. In an attempt to have a better view of the morphology of the modified eutectic Si phase, Milenkovic et al. [22] performed a deep etching on a modified Al-Si eutectic. They found that the modified eutectic Si phase consists of a network of interconnected fine coral-like fibrous structures. Accordingly, in the current work, bands of coarse Si structures were also detected along the eutectic grain boundaries, as illustrated in Figure 3. The occurrence of these coarse Si bands is accounted for by the over-modification of the eutectic Si phase [12].

**Figure 5:**
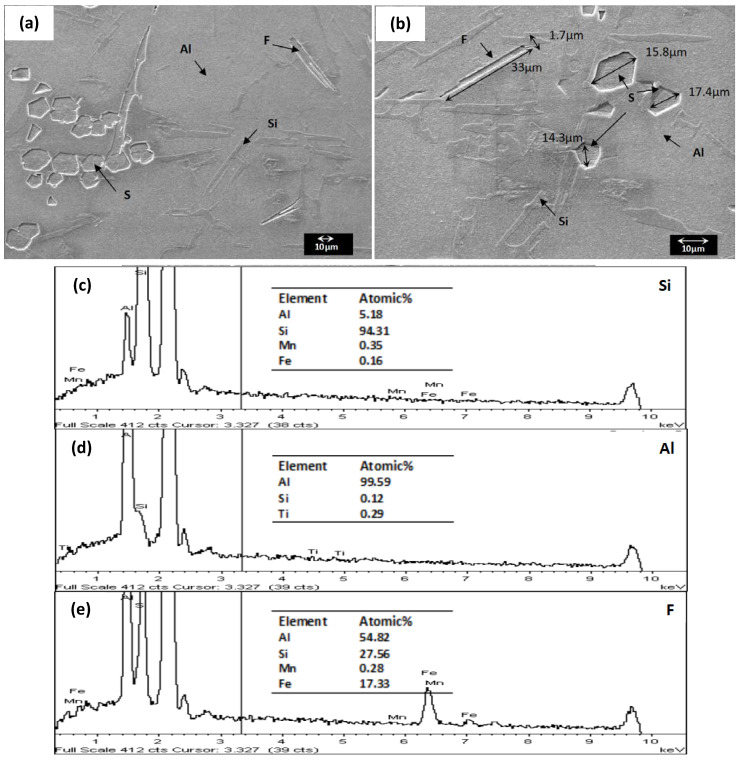
The microstructure of unmodified LM6 under SEM at (**a**) 500 (**b**) 1 k magnification 15 kV and (**c**–**e**) the corresponding EDX spectrums [F: Flake-like, S: Sludge-like, Al: Aluminium-rich phase, Si: Silicon-rich phase].

**Figure 6:**
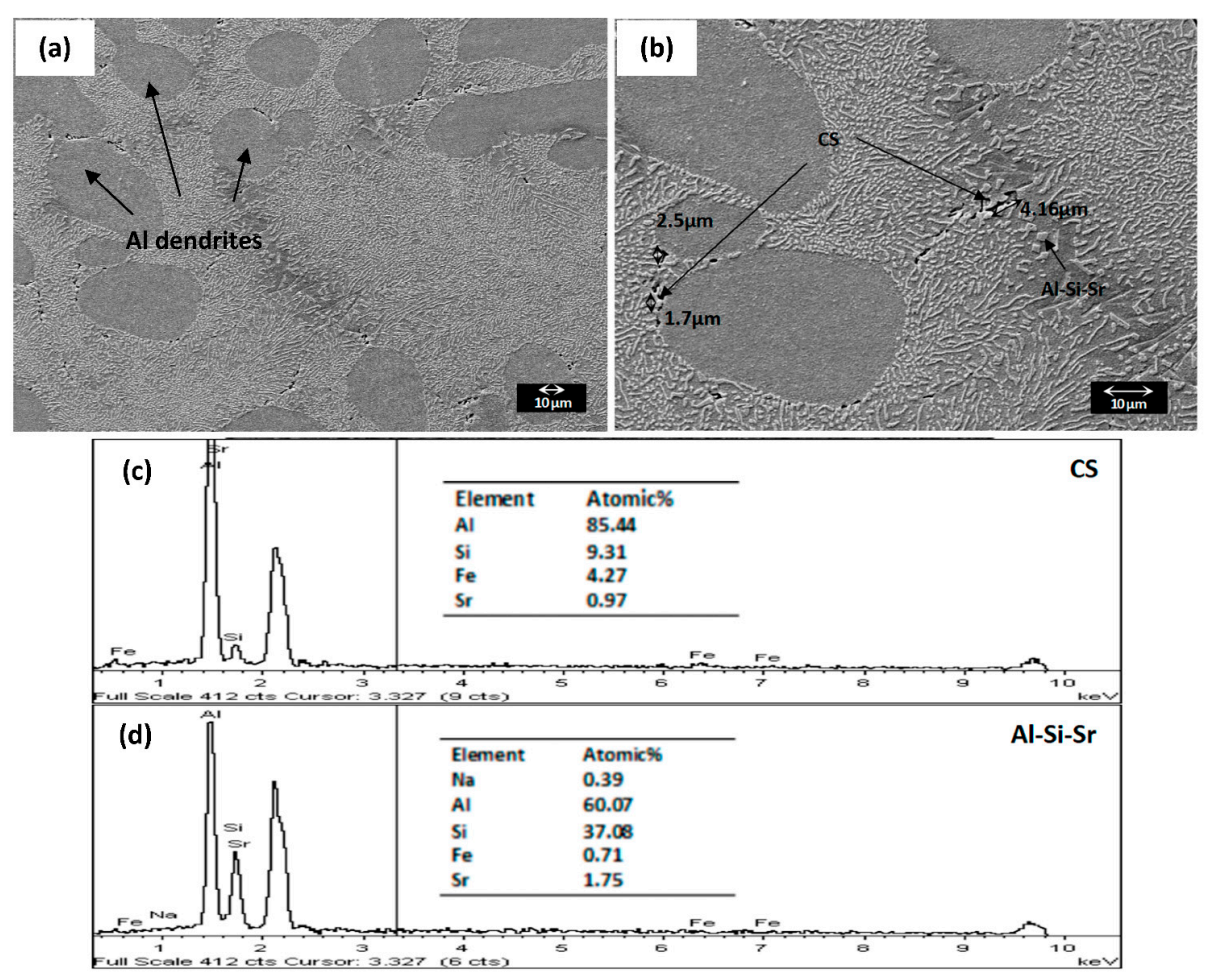
The microstructure of Na-Sr hybrid modified LM6 under SEM at (**a**) 500 (**b**) 1 k magnification 15 kV and (**c**,**d**) the corresponding EDX spectrums [Al: Aluminium, CS: Chinese Script].

**SEM-EDX.** The SEM-EDX images of the unmodified LM6 and hybrid modified LM6 at 500 and 1 k magnifications are presented in Figure 5 and Figure 6, respectively.

From Figure 6, bright needles can be observed distributed evenly across the dark matrix in the unmodified LM6. Literature has described these bright needles as coarse Si-rich plates embedded in the eutectic Al-rich matrix [7,8,23]. Based on the EDX analysis, the bright needles contain approximately 94 wt.% of Si (Figure 5c), while the dark matrix consists of 99 wt.% of Al (Figure 5d). This confirms that the bright needles and dark matrix are Si-rich needles and Al-rich eutectic matrix, respectively. Apart from the Si-rich needles and Al-rich eutectic matrix, two other phases with different morphologies, the flake (F) and the polyhedral sludge (S), can also be identified in Figure 5. The flake-like phase has a length and width of approximately 33 and 1.7µm, respectively, whereas the sludge-like phase has an average size of approximately 15.8µm (measured according to the maximum line segment). The EDX spectrum of the flake-like phase is shown in Figure 5e, revealing a significant amount of Al (55 at.%), Si (28 at.%), and Fe (17 at.%), along with a small amount of Mn (0.28 at.%). This elemental composition is close to that of the commonly reported β-Fe needles with a stoichiometry ratio of Al_5_(Fe,Mn)Si [24,25]. Biswas et al. [26] reported that the sludge-like phase has a stoichiometry composition of Al_6_(Mn,Fe). According to Ashtari et al. [27], in the presence of Mn and Fe, the solidification path of Al-Si alloy will follow the following sequence: (1) the solidification of Al-dendrites, (2) the formation of Chinese script or sludge-like α-Fe phase, and (3) the formation of needle-like β-Fe and α-Fe phases. The presence of flake-like and sludge-like Fe-bearing phases in this study supports the aforementioned viewpoint.

On the other hand, dark primary Al dendrites and bright fine fibrous eutectic Si can be observed in Figure 6 after the combined addition of Na_2_CO_3_ and Al-Sr alloy. The formation of primary Al dendrites is perhaps related to the constitutional undercooling owing to Sr that suppresses the eutectic temperature and shifts the eutectic composition to the right side of the Al-Si binary phase diagram [23]. The bright, long needle-like eutectic Si is no longer observed owing to the successful eutectic hybrid modification of Na-Sr. Besides, instead of the flake-like and sludge-like phases, fine (approximately 2–4 µm) Chinese script (CS) phase can be seen segregating at the eutectic boundary and near the Al dendrites. The segregation of Chinese script at the eutectic boundary and Al dendrites was also discovered by Timpel et al. [28]. The authors suggested that in the presence of Sr, Fe solutes will be rejected in front of the solid/liquid interface during the eutectic solidification, leading to the formation of the Fe-rich phase at the boundaries.

Additionally, Al dendrites can serve as the obstacles or preferred site of perturbation during eutectic grain growth. This results in the segregation of the Fe-rich phase at the Al dendrites. An EDX spectrum was taken at the fine Chinese script phase. Due to the fineness of the Chinese script, the EDX analysis becomes more difficult. The EDX spectrum of the Chinese script is presented in Figure 6c, revealing the peak of Al (85 at.%), Si (9 at.%), Fe (4 at.%) and Sr (1 at.%), close to the reported α-Fe phase [29]. This indicates that Na-Sr transforms the eutectic Si to fibrous morphology and suppresses the formation of β-Fe while transforming the sludge-like α-Fe to Chinese script α-Fe. The study has been reporting that the morphology of α-Fe depends on the nucleation and growth velocity [30]. Low growth velocity will produce sludge-like α-Fe, while Chinese script α-Fe favours high growth velocity. It is therefore proposed that the Na-Sr hybrid modification increases the undercooling of the Fe phase, further speeds up its growth velocity, and results in the formation of the Chinese script α-Fe phase.

Moreover, the suppression of β-Fe after adding Sr has also been reported [27,31]. It is believed that Sr can adsorb at the α-Fe/liquid interface, preventing the diffusion of Si into the α-Fe that is necessary for the formation of β-Fe. There is also another explanation given by Samuel et al. [31], proposing that the suppression of the β-Fe phase is attributed to the rejection of Si from the β-Fe needles after the addition of Sr. Al-Si-Sr phase will form at the surrounding Sr and Si-rich area. In this study, a small polyhedral phase can be identified at the eutectic grain boundary. Through EDX analysis (Figure 6d), the small polyhedral phase contains approximately 60 at.% of Al, 37 at.% of Si, 2 at.% of Sr, and 1 at.% of Fe, suggesting the presence of the Al-Si-Sr-Fe phase.

**Figure 7:**
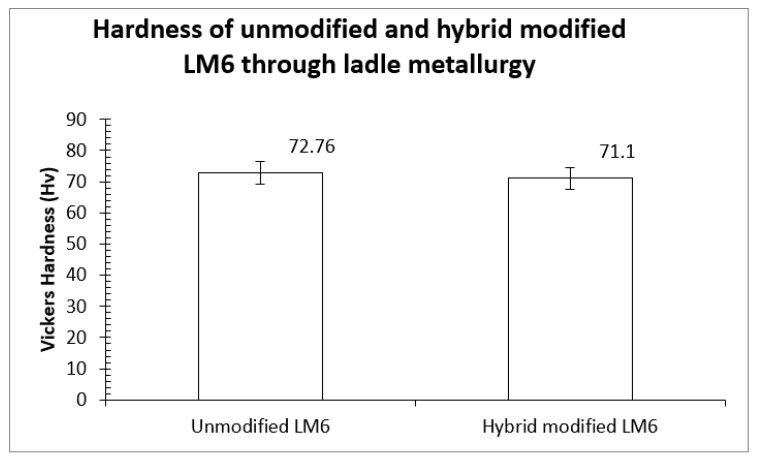
Vickers hardness of unmodified and hybrid modified LM6 with Na_2_CO_3_ and Al-10Sr alloy.

**Hardness.** Hardness provides a relatively cheap, convenient, and fast non-destructive assessment of the ability of a material to withstand plastic deformation. As highlighted in Figure 7, the hardness of LM6 decreases after the hybrid is modified by Na_2_CO_3_ and Al-Sr alloy. Before hybrid modification, 72.76 Hv was recorded. Its hardness reduces slightly to 71.10 Hv, equivalent to a 2.3% reduction after hybrid modification. Hardness reduction was also reported by [32,33] after the addition of Sr to Al-Si alloys.

The hardness reduction is believed to be very much related to the solid solution softening. The presence of Si atoms in the Al matrix has been known to increase the hardness of the Al-rich matrix through solid solution strengthening [34]. Therefore, the softening of the Al-rich matrix is most probably attributed to the incorporation of Na and Sr atoms. Besides, the hybrid-modified LM6 has not undergone any annealing or heat treatment process that relieves the strain, resulting in the softening of the Al-rich matrix. Atomic size misfit [35,36] and atomic modulus misfit [36] can be used to explain the solid solution softening results from the addition of Na and Sr atoms in an Al-rich matrix. The introduction of solute atoms having different atomic radii than that of the solvent atoms will lead to atomic size misfit. Substitution of solvent atoms by smaller solute atoms will induce compressive lattice strain, while the substitution of solvent atoms with larger solute atoms will cause tensile lattice strain. These lattice strains serve as barriers to dislocation motion and harden the solid solution. The hardness enhancement and reduction of solid solution are also governed by the shear modulus of the solute and solvent atoms. The substitution of solvent atoms by the solute atoms with shear modulus differs from that of solvent atoms. It will form local ‘hard’ and ‘soft’ spots due to the difference in elastic strain energy, leading to atomic modulus misfit [37].

To provide a quick analysis of the effects of atomic size and modulus misfits on the hardness of the Al-rich matrix, the rule of mixture [38] is applied to determine the ‘effective’ radius and shear modulus of the Al-rich matrix after the addition of Na and Sr atoms. The corresponding ‘effective’ radius and shear modulus are tabulated in Table 1. The hardness, radius and shear modulus of pure Al are also included in the table as a reference. The hardness of LM6 is greater than the hardness of pure Al due to the compressive strain induced by the smaller Si atoms, as reflected in the reduced ‘effective’ radius. Besides, the presence of Si atoms in the Al increases the ‘effective’ shear modulus. The reduction in the ‘effective’ radius and increment in ‘effective’ shear modulus resulted in the hardening of LM6. On the other hand, after the addition of Na and Sr atoms to the LM6, the ‘effective’ radius increases by 0.029% while the ‘effective’ shear modulus reduces by 0.017%. The relief of compressive strain and reduction of ‘effective’ shear modulus attributed to the presence of Na and Sr atoms leading to the reduced hardness of LM6.

However, it can be argued that atomic size misfit and modulus misfit only have slight influences in reducing the hardness of hybrid-modified LM6. The attempt to understand the reduction of hardness solely based on the atomic size and modulus misfit seems insufficient. The Na and Sr atoms may interact with vacancies, resulting in a net reduction of available defects that serve as dislocation barriers [37,40]. It was reported that a reduction in vacancy concentration can lower the hardness of an alloy. Shen and Koch [38] also discovered that the hardness of an alloy can be attributable to the combined effects resulting from the softening or hardening of solid solution and grain boundary. In this work, the reduction of hardness can also be related to the presence of the relatively soft primary dendritic Al phase in the microstructure of hybrid-modified LM6. Na and Sr atoms modify the microstructure of LM6, promoting the formation of primary Al dendrites that are not observable in unmodified LM6. This leads to the reduced volume fraction of the eutectic structure that contains hard Si particles. Similar instances were also reported in a study by Xu et al. [41]. In that study, the hardness was dependent on the percentage of the eutectic structure. The hardness reduces as the fraction of the eutectic structure decreases. Another possible reason leading to the reduction of the hardness of the hybrid-modified LM6 can be attributed to the formation of pores that serve as sites of stress concentration that subsequently reduce the hardness of LM6. The hardness reduction agrees with the decrease in density after the addition of Na_2_CO_3_ and Al-Sr alloy in this work.

**Figure 8:**
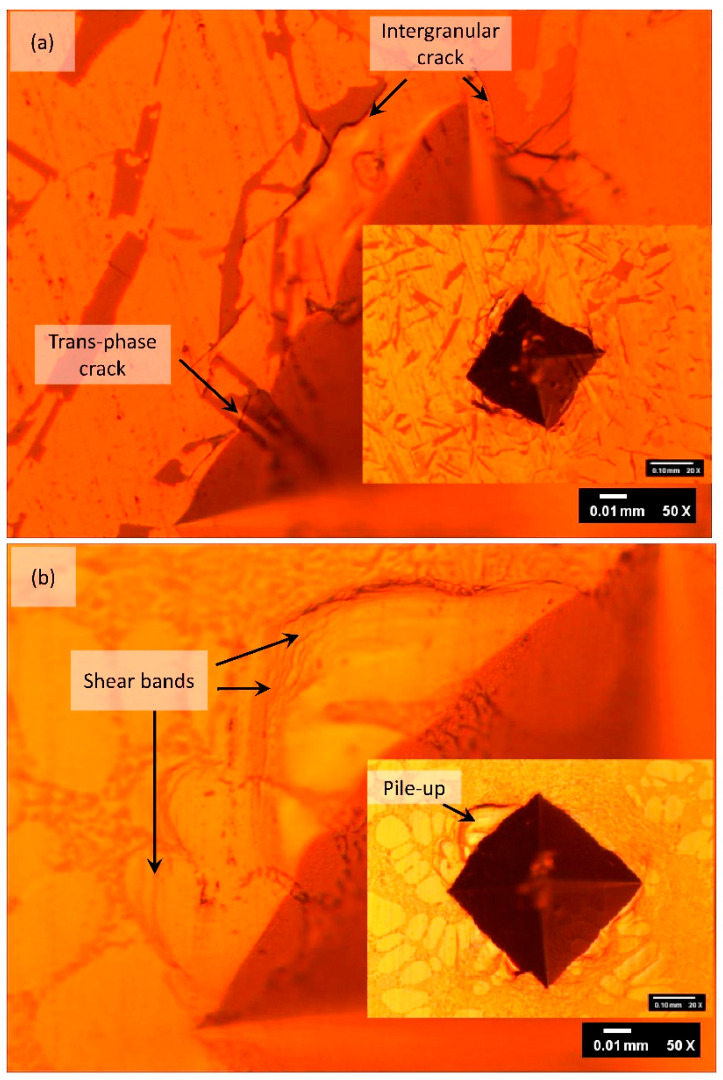
Vickers hardness indentations of (**a**) unmodified LM6 and (**b**) hybrid modified LM6 with Na_2_CO_3_ and Al-Sr alloy at 2 kg load.

To gain more insights into the mechanical behavior of hybrid-modified and unmodified LM6, the fracture features of their indentations after the hardness tests were studied. Figure 8a reveals the formation of intergranular cracks at the edge of the eutectic Si needles, indicating the brittle nature of unmodified LM6. Intra-granular cracks and trans-phase cracks were also observed. Contrarily, after hybrid modification (Figure 8b), cracks were not detected. Pile-ups of primary Al dendrites at the edge of indentation can be observed. These ‘pile-up’s indicate the hybrid-modified LM6 experienced great plastic deformation after the hardness test. The transformation of brittle to ductile fracture mode can be related to the suppression of sharp brittle β-Fe flakes and the formation of a more favourable Chinese script phase after the hybrid modification of LM6 by Na_2_CO_3_ and Al-10Sr alloy.

## 4. Conclusions

Hybrid modification of LM6 by Na_2_CO_3_ and Al-10Sr alloy through ladle metallurgy provides a means of transforming the needle-like eutectic Si to fibrous eutectic Si. However, bands of over-modification were observed along the eutectic grain boundaries. Primary Al dendrites were also formed after the hybrid modification. The hybrid modification of LM6 by Na_2_CO_3_ and Al-10Sr alloy suppressed the formation of a flake-like β-Fe phase while transforming the sludge-like α-Fe to Chinese script morphology. The density of hybrid-modified LM6 was reduced by 2.13%. A slight hardness reduction (2.3%) was recorded after the hybrid modification. Elastic atomic size and modulus misfit could not describe the hardness reduction persuasively. The increased porosity after the hybrid modification by Na_2_CO_3_ and Al-10Sr alloy might be another explanation for the hardness reduction. From the observations on the fracture surfaces of the indentations, hybrid modification by Na_2_CO_3_ and Al-10Sr alloy through ladle metallurgy can improve the ductility of LM6 through brittle to ductile transformation.

## Figures and Tables

**Table 1:** Vickers hardness, Effective Atomic Radius, and Effective Shear Modulus of Pure Al, Unmodified LM 6, and Hybrid modified LM 6.

	Vickers Hardness (Hv)	Effective Atomic Radius (nm)	Effective Shear Modulus (GPa)
Pure Al	* 17.02	* 0.14300	* 26.00
Unmodified LM 6	72.76	0.13959	33.36
Hybrid modified LM 6	71.10	0.13963	33.35

* According to data published in Materials Science and Engineering [39]. Calculations of effective atomic radius and effective shear modulus are based on data published in Materials Science and Engineering [39].
